# Editorial: Technology developments and clinical applications of artificial intelligence in neurodegenerative diseases

**DOI:** 10.3389/fneur.2026.1802039

**Published:** 2026-02-24

**Authors:** Chang Li, Chuanming Li

**Affiliations:** Medical Imaging Department, Chongqing Emergency Medical Center, Chongqing University Central Hospital, School of Medicine, Chongqing University, Chongqing, China

**Keywords:** Alzheimer's disease, artificial intelligence (AI), clinical application, cognitive impairment (CI), neurodegenerative disease, Parkinson's disease, technology development

Neurodegenerative diseases, including Alzheimer's disease (AD), Parkinson's disease (PD), multiple system atrophy (MSA), and cognitive impairment related to cerebral small vessel disease (CSVD), seriously affect global human health and impose an increasing burden on public health expenditures. The complexity of its pathogenesis, overlapping clinical manifestations, and lack of early diagnostic biomarkers make early diagnosis, differential diagnosis, and personalized intervention difficult in clinical practice. Artificial intelligence (AI) with its prowess in processing high-dimensional data and uncovering hidden mechanisms has emerged as an effective force in addressing these challenges. This Editorial synthesizes the key advancements and clinical insights from these contributing articles in this Research Topic, highlighting how AI is reshaping the diagnosis, prediction, subtyping, and treatment of neurodegenerative diseases.

Artificial intelligence can provide objective, non-invasive, and intelligent tools to help distinguish early diagnosis, differential diagnosis, and subtype differentiation of neurodegenerative diseases. Zhang et al. conducted a comprehensive 25 years bibliometric analysis that revealed exponential growth in AI applications after 2017 and identified neuroimaging analysis and innovations in machine learning methodologies as key research hotspots. This macro perspective is complemented by targeted clinical studies. For posterior circulation stroke (PCS), Hassan et al. developed an eye-tracker-based AI tool that incorporates three oculomotor assessments: the Dot Test, H Test, and Optokinetic Nystagmus (OKN) Test. This machine learning-powered tool achieved 96% sensitivity and 88% accuracy, underscoring its potential to enable more accurate and efficient diagnosis, particularly for providers without neurology training, and improved patient outcomes via timely targeted interventions. Xie et al. integrated Fast Dixon with deep learning denoising technology to enhance SNR and CNR of brachial plexus MRI without contrast agents, improving visualization of fine neural structures. This non-invasive high-quality imaging approach provided technical support for early detection of neural lesions. Subtyping of neurodegenerative diseases, which is crucial for personalized treatment, has also benefited from AI advancements. Hui et al. used radiomics features from 8 deep brain nuclei and five machine learning classifiers, with the Bagging Decision Tree model achieving an AUC of 0.962 in distinguishing PD's tremor-dominant (TD) and postural instability/gait difficulty (PIGD) subtypes. Lu et al. further advocated for multimodal imaging fusion encompassing MRI, PET, and CT paired with end-to-end AI models, emphasizing that integrating structural, metabolic, and functional data yields higher diagnostic accuracy than single-modal strategies. Li et al. developed a radiomics model using multimodal MRI sequences including T1WI, T2WI, FLAIR, and DWI, achieving 95% accuracy in distinguishing multiple system atrophy (MSA) patients from healthy controls and identifying the left putamen as the most influential predictor.

Beyond the application in cross-sectional diagnosis, artificial intelligence has demonstrated extraordinary abilities in disease prediction. This capability encompasses forecasting disease onset years before clinical symptoms manifest, predicting the progression rate in affected individuals, and identifying the conversion risk from prodromal stages such as mild cognitive impairment to full-blown neurodegenerative disease, thereby addressing a longstanding clinical need for proactive intervention. Zeng et al. leveraged multimodal data including neuroimaging, clinical, and biological markers to develop an AI model for early AD prediction, which enabled risk stratification years before symptom onset. Meanwhile, Ai et al. systematically synthesized the progress of multimodal MRI in capturing brain alterations associated with MCI-to-AD conversion, alongside the application of AI algorithms in developing robust prediction models. The study analyzed the current technical challenges in this field and outlined future research directions, aiming to provide a scientific foundation for the early, accurate prediction of MCI conversion and the development of targeted intervention strategies.

AI's role in identifying novel biomarkers and unraveling disease mechanisms is also important. Liu et al. conducted a 3-month longitudinal study on common-type COVID-19 patients, revealing acute-phase morphological brain network disruptions and partial recovery thereafter including FPN-LN connectivity restoration, SCN compensation, and improved cognitive function. This study underscored the value of AI-driven longitudinal analysis in tracking progression and identifying recovery biomarkers. Zheng et al. used tract-based spatial statistics (TBSS) on diffusion tensor imaging (DTI) data, constructing a multivariate logistic regression model that effectively identified cognitive impairment in CSVD patients, linking specific white matter tract damage to cognitive decline. Complementing this, Du et al. noted that white matter hyperintensity (WMH) served as a core imaging biomarker for CSVD, with pathogenesis involving hypoperfusion and blood-brain barrier disruption. Radiomics integrated with multiple deep learning algorithms enabled the extraction of microstructural features from WMH, offering a non-invasive tool to identify potential biomarkers and elucidate underlying pathological mechanisms. Li et al. used the Random Forest model to integrate structural MRI features, neuropsychological assessments, and laboratory data, identifying 8 optimal biomarkers for the diagnosis of MCI in patients with type 2 diabetes mellitus. This work underscored the interplay between metabolic dysregulation and neurodegeneration in MCI pathogenesis. In AD research, Stramba-Badiale et al. systematically reviewed autobiographical memory deficits in AD, marked by reduced memory specificity, altered temporal gradients, and links to hippocampal and prefrontal cortex impairment. These deficits acted as potential early biomarkers of disease progression, while sensory cues like music and odors offered clinical value in aiding memory retrieval.

In addition, Fang et al. also emphasized the role of artificial intelligence in the study of neural mechanisms of brain diseases, and pointed out that the multimodal integration of neural networks, neuroimaging, multi-omics, and clinical records provides assistance in elucidating disease mechanisms. These technologies can accelerate the discovery of new drugs and promote personalized closed-loop therapy by discovering new targets. However, challenges including model interpretability, data standardization, and clinical validation must be addressed for widespread clinical adoption. Meanwhile, another study further explored this area, offering additional insights into AI's transformative impact on brain diseases. Wen et al. focused on transforming long-term adjunctive therapy for cognitive impairment, proposing a multimodal self-adaptive digital medicine framework that integrates multimodal interventions, self-adaptive systems and digital medicine. Leveraging AI, this framework analyzes real-time neural activity, behavioral patterns and physiological parameters to dynamically optimize treatment regimens. It forms a closed-loop feedback cycle, continuously adjusting interventions such as cognitive training, brain stimulation and behavioral support based on patients' evolving needs. This integration effectively bridges the gap between diagnostic insights and therapeutic action, realizing personalized and sustainable treatment. Such advancements highlight AI's transition from a mere diagnostic tool to a central driver of precision neurotherapeutics, offering great potential to improve long-term cognitive function and quality of life for patients with cognitive impairment.

Despite these advancements, critical challenges still remain. The bibliometric analysis by Zhang et al. underscored the need for enhanced interdisciplinary collaboration, advanced deep learning models, integration of bioinformatics and multi-omics approaches, explainable AI for clinical decision support, early detection using digital biomarkers, and multimodal data. Issues of model interpretability such as the “black-box” problem, standardized data collection across institutions, and ethical considerations were also highlighted by Lu et al. Additionally, while cross-sectional studies dominate current research, Liu et al. emphasized the need for more longitudinal investigations to capture dynamic disease trajectories, ensuring that biomarkers and diagnostic tools remain reliable over time. As highlighted in multiple studies, future directions include advanced deep learning architectures, multi-omics integration, explainable AI systems, digital biomarker-based early detection, AI-driven predictors, and closed-loop therapeutic platforms.

In summary, the contributing articles collectively demonstrate that AI is no longer a theoretical concept but a clinically actionable tool across the full spectrum of neurodegenerative disease research. These studies span from macro-level landscape mapping to micro-level biomarker discovery, and from early prediction of disease onset and progression to personalized treatment and closed-loop therapeutics. Leveraging longitudinal data, specialized imaging sequences, and multimodal analytics, AI is accelerating progress in key areas including diagnosis, subtyping, prediction, mechanism elucidation, and therapeutic innovation. With the continuous advancement of AI technology and its deep integration into clinical practice, addressing current limitations through standardized data sharing, interdisciplinary collaboration, and model explainability will remain pivotal to bridging the gap between technological innovation and clinical applications. This Research Topic stands as a testament to AI's transformative role in neurodegenerative disease research reflecting its recent advancements and clinical applications. It paves the way for a more precise, efficient, and patient-centered paradigm where technological developments synergize with clinical needs to narrow the gap between AI innovations and practical applications. This redefines disease management from enhanced early diagnosis and improved prognostic evaluations to timely interventions and long-term care, unlocking AI's full potential to improve outcomes for patients worldwide.

